# Calebin A Potentiates the Effect of 5-FU and TNF-β (Lymphotoxin α) against Human Colorectal Cancer Cells: Potential Role of NF-κB †

**DOI:** 10.3390/ijms21072393

**Published:** 2020-03-31

**Authors:** Constanze Buhrmann, Ajaikumar B. Kunnumakkara, Bastian Popper, Muhammed Majeed, Bharat B. Aggarwal, Mehdi Shakibaei

**Affiliations:** 1 Musculoskeletal Research Group and Tumour Biology, Chair of Vegetative Anatomy, Institute of Anatomy, Faculty of Medicine, Ludwig-Maximilian-University Munich, Pettenkoferstrasse 11, D-80336 Munich, Germany; constanze.buhrmann@med.uni-muenchen.de; 2 Cancer Biology Laboratory & DBT-AIST International Laboratory for Advanced Biomedicine (DAILAB), Department of Biosciences & Bioengineering, Indian Institute of Technology Guwahati, Assam 781039, India; kunnumakkara@iitg.ac.in; 3 Biomedical Center, Core facility animal models, Ludwig-Maximilian-University Munich, D-82152 Martinsried, Germany; Bastian.Popper@bmc.med.lmu.de; 4 Institute of Pathology, School of Medicine, Technical University of Munich, D-81675 Munich, Germany; 5 Sabinsa Corporation, East Windsor, NJ 08520, USA; mmjd52@hotmail.com; 6 Inflammation Research Center, San Diego, CA 92126, USA; bbaggarwal@gmail.com

**Keywords:** Calebin A, colorectal cancer, TNF-β (lymphotoxin), 5-Fluorouracil, chemosensitization, NF-κB

## Abstract

Objective: The majority of chemotherapeutic agents stimulate NF-κB signaling that mediates cell survival, proliferation and metastasis. The natural turmeric non-curcuminoid derivate Calebin A has been shown to suppress cell growth, invasion and colony formation in colorectal cancer cells (CRC) by suppression of NF-κB signaling. Therefore, we hypothesized here that Calebin A might chemosensitize the TNF-β-treated tumor cells and potentiates the effect of 5-Fluorouracil (5-FU) in advanced CRC. Materials and Methods: CRC cells (HCT116) and their clonogenic 5-FU chemoresistant counterparts (HCT116R) were cultured in monolayer or alginate-based 3D tumor environment culture and were treated with/without Calebin A, TNF-β, 5-FU, BMS-345541 and DTT (dithiothreitol). Results: The results showed that TNF-β increased proliferation, invasion and resistance to apoptosis in chemoresistant CRC cells. Pretreatment with Calebin A significantly chemosensitized HCT116R to 5-FU and inhibited the TNF-β-induced enhanced efforts for survival, invasion and anti-apoptotic effects. We found further that Calebin A significantly suppressed TNF-β-induced phosphorylation and nuclear translocation of p65-NF-κB, similar to BMS-345541 (specific IKK inhibitor) and NF-κB-induced tumor-promoting biomarkers (NF-κB, β1-Integrin, MMP-9, CXCR4, Ki67). This was associated with increased apoptosis in HCT116 and HCT116R cells. Furthermore, blocking of p65-NF-κB stimulation by Calebin A was imparted through the downmodulation of p65-NF-κB binding to the DNA and this suppression was turned by DTT. Conclusion: Our findings indicate, for the first time, that Calebin A chemosensitizes human CRC cells to chemotherapy by targeting of the p65-NF-κB signaling pathway.

## 1. Introduction

Human colorectal cancer (CRC) is the third most frequent tumor worldwide, one of the most commonly diagnosed cancers and the main reason for tumor-related morbidity and mortality [1]. Chemotherapy coupled with surgery is the major treatment option for metastatic CRC. Among chemotherapeutic options, 5-Fluorouracil remains the most effective option since its discovery and is included most frequently in the treatment protocols [2,3,4]. However, it is well known that chemotherapeutic treatment increases chemoresistance and poses a major therapeutic challenge [5]. The main reason for this is an adaptation of the cancer cells to conventional chemotherapy drugs, resulting in relapses, metastasis and death. For this reason, there is a huge necessity to develop pharmacologically-safe and relatively inexpensive anti-tumor agents as potential adjunctive treatments to enhance the overall treatment response of patients with CRC [6,7].

It is well known that chronic low-grade inflammation plays a major role in mediating cancer proceedings in several cancers [8,9]. Nuclear transcription factor NF-κB, as a central responder to stress signaling, plays a fundamental role in the inflammatory cascade [10,11]. Through autocrine and paracrine signaling in the tumor and the tumor environment, NF-κB may be activated by a variety of stimuli including inflammatory cytokines and growth factors promoting cancer cell survival, proliferation, invasion and metastasis [11,12]. Pro-inflammatory cytokines belonging to the Tumor Necrosis Factor family, such as TNF-α and TNF-β, play a key role in chronic diseases such as cancer [13]. TNF-β, the closest structural homolog to TNF-α, was identified around 35 years ago [14,15] and has recently come to attention as it may stimulate NF-κB activation in cancer cells with a similar potency to TNF-α, thereby up-regulating proliferation, invasion and malignancy of cancer cells [16,17,18].

Additionally, it has been described that chemotherapeutic agents stimulate the activation of nuclear transcription factor NF-κB, thus enhancing chemoresistance mechanisms in cancer cells [19,20,21].

Extensive investigations have demonstrated that several natural agents have chemopreventive/chemosensitization and therapeutic potency against many chronic diseases including cancer [22]. A large body of evidence has shown that numerous plants have solid pharmacological characteristics and, indeed, over 60% of all drugs used in western medicine are originally derived from natural sources [23,24]. Turmeric rhizome (*Curcuma longa L*. Zingiberaceae) is one of the most traditionally executed medicinal plant-derived products in the traditional medicine applied in India and China. Several lines of evidence have shown that turmeric has a wide array of biological properties, including anti-cancer, anti-inflammatory, anti-oxidant, anti-osteoarthritis, anti-aging, anti-microbial and wound healing [25,26]. In the past, the majority of investigations on the field of turmeric were focused primarily on its major polyphenolic ingredient curcumin [27]. However, recently attention has come to Calebin A, another type of turmeric rhizome pharmacologically active component [18,25,28]. The active compound Calebin A is an ingredient isolated from curcumin-free turmeric extract and has been reported to present anti-tumor qualities by targeting and suppression of a variety of molecular signaling pathways including the NF-κB pathway [18,28,29,30].

Although cytokine-induced NF-κB activation is well known to promote chemoresistance of cancer cells to 5-Fluorouracil (5-FU), the potential chemosensitization effect of Calebin A on CRC cells has not been investigated. Therefore, in the present investigation, we hypothesized that Calebin A could modulate and sensitize 5-FU resistant and non-resistant CRC cells in the TNF-β-promoted inflammatory tumor environment in monolayers and 3D-alginate culture model.

## 2. Results

The goal of this paper was to investigate whether Calebin A might have a role in the induction of the chemosensitization effects on wild-type and 5-FU chemoresistant cancer cells to chemotherapeutic agents, by targeting proteins involved in cell survival, proliferation, metastasis and apoptosis in a TNF-β-mediated inflammatory environment. We used a well-characterized CRC cell line (parental HCT116) and their clonogenic 5-FU chemoresistant counterparts (HCT116R) to address various questions in the monolayer and 3D alginate-based tumor environment.

### 2.1. Calebin A Chemosensitizes and Potentiates the Effect of 5-FU to CRC cells and Inhibits Cell Proliferation in TNF-β-Stimulated Tumor Environment Cultures

**Figure 1:**
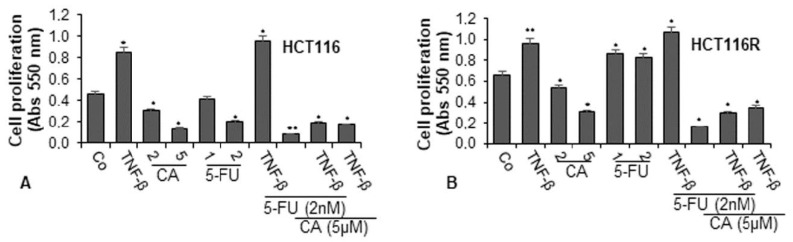
Effects of Calebin A and/or 5-Fluorouracil (5-FU) on TNF-β-promoted cell proliferation in colorectal cancer cells (CRC) cells in the monolayer culture. Serum-starved cultures of HCT116 (**A**) and HCT116R (**B**) cell lines were treated as described in the Materials and Methods section. Cell viability and proliferation were evaluated with the MTT method. All assays were performed at least three times. *p* < 0.05 (*) and *p* < 0.01 (**) indicate a significant difference compared to the control group.

To examine the effect of Calebin A and/or 5-FU on TNF-β-promoted cell viability and proliferation of CRC cells (HCT116 and HCT116R) in the monolayer of the inflammatory tumor environment, the cells were treated as described in detail in the Materials and Methods section and the capacity of proliferation was assessed by MTT assay. TNF-β significantly promoted the proliferation of both CRC cells by almost 100% in HCT116 and around 50% in HCT116R cells compared to control (Figure 1A,B). These findings underline that TNF-β markedly induced HCT116 and HCT116R cell proliferation. In contrast, Calebin A (2, 5 µM) suppressed the cell viability and proliferation of both tumor cells in a dose-dependent manner in HCT116 cells by 33% and 89% and in HCT116R cells by 20% and 53%, respectively. Treatment of the CRC cells with 5-FU (1, 2 nM) by itself blocked proliferation of HCT116 cells in a dose-dependent manner by 9% and 56%, respectively (Figure 1A). As reported earlier, there was no effect of 5-FU on the 5-FU resistant cells (HCT116R), even after treatment with 2 nM dose (Figure 1B). On the contrary, treatment with 5-FU increased cell proliferation by around 30% in HCT116R cells compared to untreated control. Moreover, we found that the common treatment of 5-FU with TNF-β synergistically increased the proliferation capacity of HCT116 (around 5%) and HCT116R (around 10%) cells (see above) more than each agent by itself (Figure 1A,B). However, HCT116R cells proliferated more under the same condition compared to HCT116 cells, suggesting that TNF-β created an inflammatory tumor environment under chemotherapeutic treatment and upregulated the malignant capacity of CRC cells for the 5-FU resistant cells (Figure 1). Moreover, we examined whether Calebin A downregulates the increased proliferation of the CRC cells through co-treatment of 5-FU and/or TNF-β. As shown in Figure 1A,B, we found that treatment of CRC cells (HCT116 and HCT116R) with Calebin A (2, 5 µM) by itself (*p* < 0.05) or as co-treatment with 5-FU (2 nM) and/or TNF-β (10 ng/mL) at Calebin A (5 µM) suppressed the proliferation capacity of HCT116 and HCT116R cells significantly by around 50% compared to untreated cells (Figure 1A,B). Taken together, these findings suggest that TNF-β can promote and induce tumor cell activation and proliferation, thereby enhancing the malignancy of the cancer cells. Suppression of this pro-inflammatory pathway by Calebin A promotes signaling changes towards sensitizing CRC cells to 5-FU treatment.

### 2.2. Calebin A Downmodulates TNF-β-Induced Colonosphere Formation and Migration in CRC Cells in 3D Cultures

**Figure 2:**
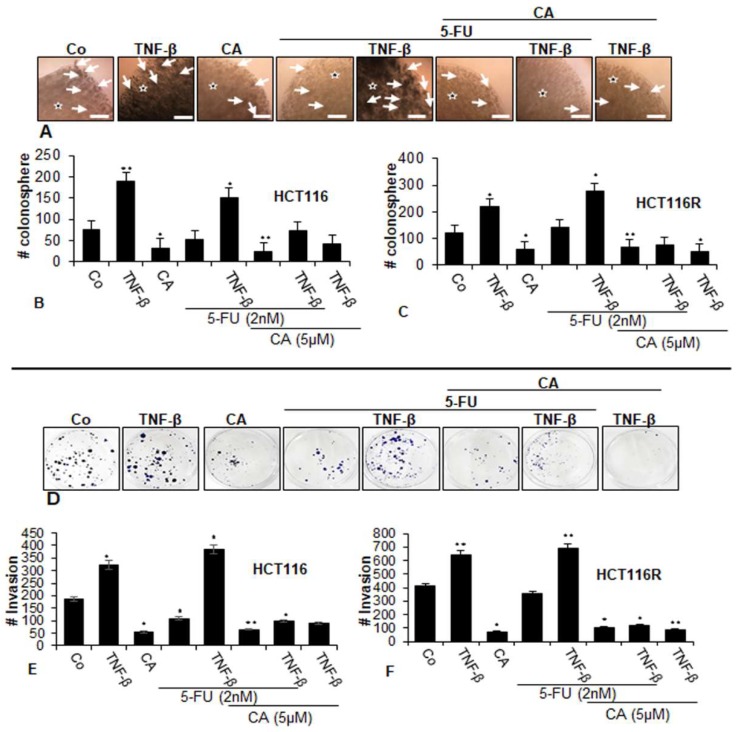
Effects of Calebin A and/or 5-FU on TNF-β-promoted colonosphere formation and migration in CRC cells in 3D-alginate tumor cultures. Serum-starved cultures of HCT116 (**A**,**B**,**D**,**E**) and HCT116R (**C**,**F**) cell lines in alginate matrix (stars) were treated as described in the Materials and Methods section. Colonosphere formation and migration were evaluated by light microscopy after 10 days. All experiments were performed at least three times. The number of colonospheres (arrows) was quantified by counting 100 cells from 20 different microscopic fields, and the number of invaded spheroids was quantified in each well. *p* < 0.05 (*) and *p* < 0.01 (**) indicate a significant difference compared to the control group. Magnification A: ×24, scale bar = 0.2 mm.

To examine the differential activity of the Calebin A, we next evaluated whether Calebin A and/or 5-FU inhibited the capacity of two CRC cell lines (parental HCT116 and chemoresistant HCT116R) for colonosphere formation (Figure 2A–C) and to suppress migration (Figure 2D–F) in TNF-β-induced tumor environments using phase-contrast light microscopy. As shown in Figure 2, TNF-β, increased the number of colonosphere formations and migrations significantly in HCT116 and HCT116R cells compared to that in control cultures (Figure 2A–F), underlining the critical role of TNF-β-mediated inflammatory environment in promoting malignant potential of CRC cells. Treatment with Calebin A alone downregulated colonosphere formation and migration of both CRC cell lines in alginate culture (Figure 2A–F). Treatment of both CRC cell lines with 5-FU by itself blocked colonosphere formation and migration in HCT116 cells but not in HCT116R cells in alginate cultures; however. this was not significant (Figure 2A–F). Furthermore, we found that the combined treatment of 5-FU with TNF-β, similar to TNF-α, synergistically enhanced the colony formation and migration capacity of HCT116 and HCT116R cells in comparison to each agent by itself (Figure 2A,F). Furthermore, in the presence of Calebin A and/or TNF-β both CRC cell lines showed a strongly reduced number of colonosphere formations and migrations in both CRC cell lines (Figure 2A,F). Next, we evaluated whether Calebin A modulates the colonosphere formation and migration of the CRC cells (HCT116 and HCT116R) by combined treatment with 5-FU and/or TNF-β in 3D alginate-based culture tumor environment. As shown in Figure 2, we found that treatment with Calebin A (5 µM) by itself (*p* < 0.05) and/or combination with 5-FU (2 nM) and TNF-β (10 ng/mL) strongly blocked the colonosphere formation and migration capacity of HCT116 and HCT116R cells in the alginate-based matrix compared to untreated control cells (Figure 2A–F). Taken together, these findings underline that TNF-β as an inflammatory cytokine can stimulate CRC cells to proliferate and migrate, enhancing malignancy of the tumor cells. Suppression of this inflammatory signaling pathway by Calebin A modulates signaling changes towards sensitizing CRC tumor cells to 5-FU treatment.

### 2.3. Calebin A Promotes Apoptosis in CRC cells

**Figure 3:**
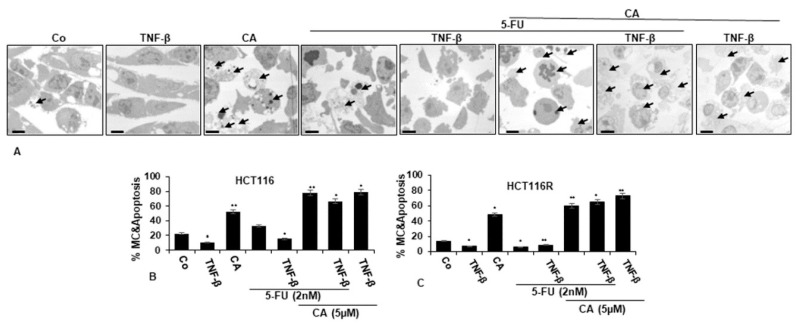
Transmission electron microscopic demonstration of cell viability and apoptosis of CRC cells after treatment with Calebin A and/or 5-FU in TNF-β-promoted inflammatory tumor environment. Serum-starved HCT116 (**A**,**B**) and HCT116R (**C**) in monolayer cultures were treated as described in the Materials and Methods section for 72 h, and ultrastructural investigations performed to investigate cell survival and apoptosis (black arrows). The number of apoptotic cells (**B**,**C**) was quantified by counting 800–1000 cells from 30 microscopic fields from at least three independent experiments. *p* < 0.05 (*) and *p* < 0.01 (**) were regarded as statistically significant values compared to control cultures. A: Magnification: ×5000, scale bar = 1 μM. MC: mitochondrial changes.

To understand the mechanistic coherence of Calebin A and its anti-tumorigenic effect in TNF-β-promoted inflammatory tumor environment and/or 5-FU in monolayer cultures, the ultrastructural investigation was conducted. CRC cells (HCT116 and HCT116R cells) proliferated well in control monolayer cultures and the cells contained mitochondria, multiple cell organelles and intact nuclei (Figure 3A–C). Similar to untreated control cultures, TNF-β-treated cells were alive, revealing an active and bipolar morphology with multiple cell organelles and well-developed nuclei (Figure 3A–C). Treatment of CRC cells with Calebin A or 5-FU by itself revealed degeneration of cell organelles, mitochondrial swelling and appearance of multiple vacuoles, with prominent evidence of typical apoptotic bodies, while these impacts were not apparent in 5-FU treated HCT116R cells (Figure 3A–C). Furthermore, we found that the co-treatment of 5-FU with TNF-β enhanced the cell viability impact of HCT116 and HCT116R cells in comparison to each agent by itself (Figure 3A–C). However, as shown in Figure 3, co-treatment of HCT116 and HCT116R cells with 5-FU and Calebin A markedly increased the degeneration of both CRC tumor cell lines. More interestingly, the co-treatment of Calebin A and/5-FU and/or TNF-β inhibited stimulation effects of TNF-β on CRC cell lines vitality and enhanced apoptosis in both CRC cells (Figure 3A–C).

Ultrastructure changes highlighted the apoptotic effects of Calebin A, 5-FU or co-treatment with TNF-β compared to control cultures (Figure 3B–C) and suggests that Calebin A may sensitize 5-FU in the parental HCT116 and chemoresistant HCT116R cell line. These results indicate a potential treatment option for Calebin A and 5-FU resistant colon cancer cells.

### 2.4. Calebin A Downmodulates TNF-β- or 5-FU-Induced Nuclear Translocation of p65-NF-κB in CRC Cells

**Figure 4:**
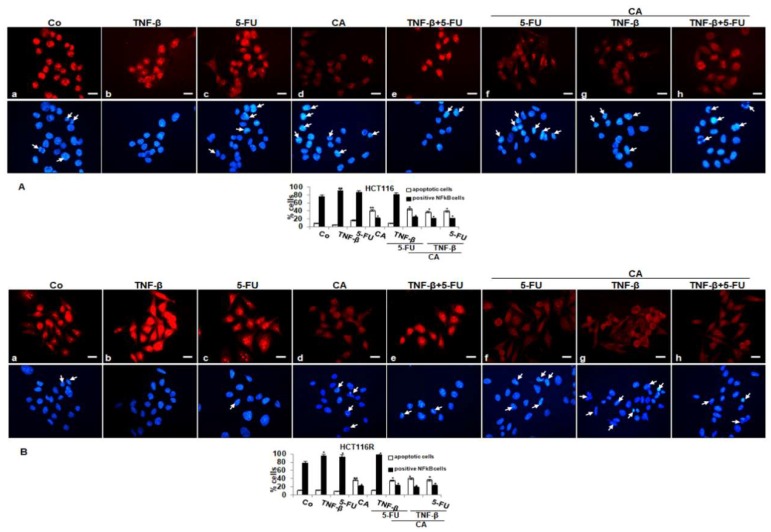
Effect of Calebin A and/or 5-FU on TNF-β-promoted activation and nuclear translocation of p65-NFκB in CRC cells in monolayer tumor cultures. Serum-starved HCT116 (**A**) and HCT116R (**B**) cells in the monolayer culture were treated as described in the Materials and Methods section, labeled for p65 by immunofluorescence and counterstained with DAPI. All experiments were performed at least in triplicate and quantification of positively labeled p65-NF-κB-nuclei and apoptotic nuclei (white arrows) were performed by counting 500–800 cells from 20 different microscopic fields. Values were compared with the control. *p* < 0.05 (*) and *p* < 0.01 (**) were considered statistically significant. Magnification 400×; scale bar = 30 nm

To determine signal transduction pathways participating in the anti-inflammatory actions of Calebin A, we examined transcription factor NF-κB linked with malignity and survival of CRC cells (HCT116, HCT116R) and performed immunofluorescence labeling for p65-NF-κB as described in the Materials and Methods section. The untreated cells revealed intense nuclear staining and weak cytoplasmic staining in HCT116 (85%) and HCT116R (79%) cells (Figure 4A,B). Treatment with TNF-β or 5-FU activated p65-NF-κB and nuclear staining strongly enhanced to 90% and 87% in HCT116 and to 89% and 83% in HCT116R. Treatment with Calebin A showed markedly fewer amounts of nuclear staining in HCT116 22% and 25% in HCT116R, respectively. Co-treatment with TNF-β and 5-FU showed a synergistically enhanced nuclear labeling in HCT116 to 82% and 91% in HCT116R, respectively. Treatment with Calebin A in the presence of TNF-β, 5-FU or TNF-β followed by 5-FU, markedly reduced nuclear labeling in HCT116 to 21%, 25% and 21% and in HCT116R to 21%, 27% and 27%, respectively. Taken together, these findings suggest that modulatory actions on proliferation, migration and colonosphere formation in CRC cells by Calebin A are mediated, at least, in part, through the suppression of the NF-κB signaling pathway. Moreover, Calebin A has the capacity to modulate the cancerogenic activation effects promoted by inflammatory cytokine TNF-β.

In addition, minimal apoptotic changes were observed in untreated cells or treated with TNF-β or 5-FU or TNF-β and 5-FU (by 9%, 5%, 14% and 9% in HCT116 and by 10%, 11%, 8% and 11% in HCT116R cells, respectively) as seen by DAPI staining. Furthermore, we found significantly more apoptotic cells in CRC treated with Calebin A by itself or treated with Calebin A and with TNF-β or 5-FU or TNF-β and 5-FU in HCT116 (42%, 36%, 45% and 40%, respectively) or HCT116R cells (39%, 40%, 35% and 38%, respectively). Altogether, these results are consistent with the body of evidence from other results that inhibition of NF-κB by Calebin A could play an important role in the prevention of tumor.

### 2.5. Calebin A Downmodulates NF-κB Activation and NF-κB-Dependent Gene Products Involved in Migration, Metastasis and Apoptosis of CRC Cells

Next, we examined and analyzed the mechanism of Calebin A-suppressed TNF-β-promoted malignancy of CRC cells and how Calebin A chemosensitizes CRC cells to chemotherapeutic agent 5-FU. We probed whether the actions of Calebin A on CRC cells in TNF-β-promoted pro-inflammatory tumor environments were linked with the suppression of NF-κB activation and NF-κB-dependent gene products involved in cancer cell metastasis.

**Figure 5:**
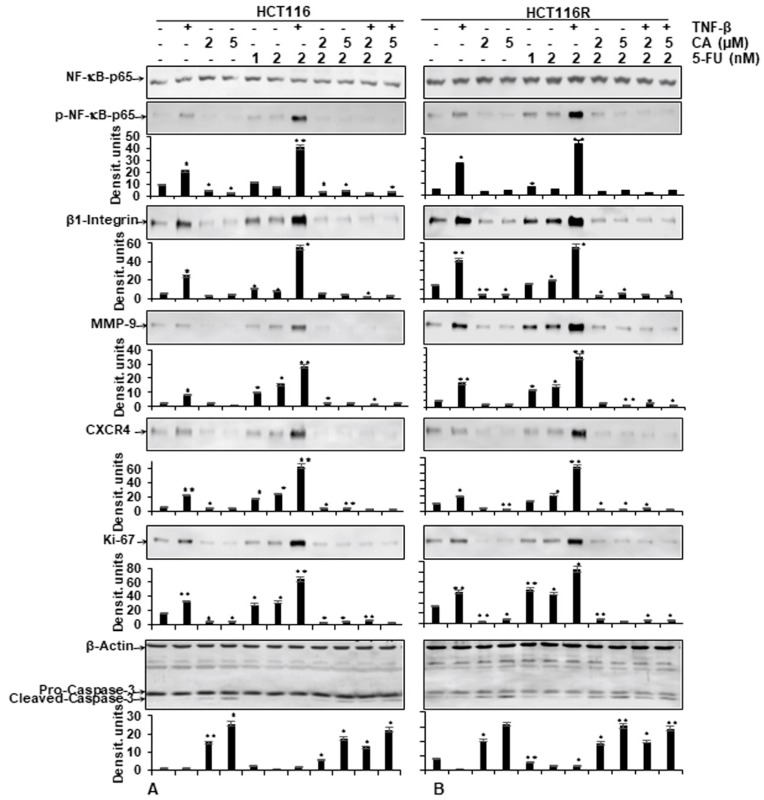
Effect of Calebin A and/or 5-FU on TNF-β-promoted p65-NFκB activation and NF-κB-regulated gene end-products involved in apoptosis, proliferation and metastasis promoted by TNF-β in CRC cells Immunoblotting of whole-cell lysates from HCT116 (**A**) and HCT116R (**B**) in monolayer cultures treated as described in the Materials and Methods section was performed for anti-p65-NF-κB, anti-phospho-p65-NF-κB, anti-β1-integrin, anti-MMP-9, anti-CXCR4, anti-Ki67 and anti-cleaved-caspase-3. β-actin served as an internal loading control in all experiments. For densitometric evaluation, results are compared to control. *p* < 0.05 (*) and *p* < 0.01 (**) were considered statistically significant.

As shown in Figure 5, the protein expression of the NF-κB activation and NF-κB-promoted gene products involved in invasion (MMP-9), metastasis (CXCR4, β1-integrin) and proliferation (Ki-67) were strongly enhanced in the presence of TNF-β, similar to 5-FU treatment by itself, or in combination treatment (TNF-β with 5-FU) in both CRC cells (HCT116, HCT116R) (Figure 5A,B). In contrast, the immunoblotting analysis showed that Calebin A by itself or in combination with 5-FU or with TNF-β and 5-FU downregulated the mentioned proteins expression in both CRC cells (Figure 5A,B). Altogether, these results were in accordance with the results observed by immunofluorescence methods. Further, these results indicate the important role of Calebin A in downmodulating TNF-β and/or 5-FU-induced NF-κB-promoted cancer metastasis gene products in CRC cells. We further examined whether Calebin A can modulate NF-κB-dependent gene products associated with apoptosis (cleavage of caspase-3) in TNF-β- and/or 5-FU-treated CRC cells. As shown in Figure 5A,B, Calebin A clearly promoted caspase-3 cleavage in HCT116 and HCT116R. The co-treatment with Calebin A and 5-FU and/or with TNF-β showed synergistic enhancement in promoting caspase-3 cleavage in both CRC cells (Figure 5A,B) compared to control tumor cultures, indicating that Calebin A enhanced TNF-β/5-FU-promoted caspase-3 dependent apoptosis in CRC cells. Taken together, these results underline the important role of Calebin A in modulating TNF-β- and/or 5-FU-promoted NF-κB-regulated biomarkers.

### 2.6. Calebin A, Similar to IKK Inhibitor (BMS-345541), Specifically Blocks TNF-β- and/or 5FU-Induced p65-NF-κB Phosphorylation in CRC Cells

**Figure 6:**
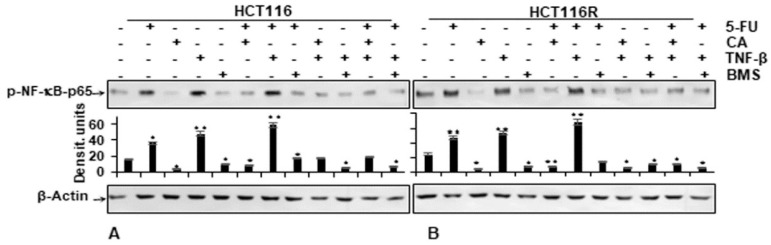
Effect of Calebin A or BMS-345541 and/or 5-FU on TNF-β-promoted p65-NFκB activation in CRC cells Serum-starved monolayer cultures of HCT116 (**A**) and HCT116R (**B**) were treated as described in the Materials and Methods section and whole-cell lysates immunoblotted for phospho-p65-NF-κB. β-actin was used as an internal loading control. Densitometric values were compared with the control. *p* < 0.05 (*) and *p* < 0.01 (**) were considered statistically significant.

We examined further up-stream cascades in the NF-κB signaling pathway to explain the effect of Calebin A. HCT116 and HCT116R cells were left untreated or treated as indicated in the Materials and Methods section. Immunoblotting findings in Figure 6 indicate that Calebin A has the potential to downmodulate TNF-β-promoted p65-NF-κB phosphorylation with the same effect as BMS-345541 in CRC cells (Figure 6A,B). The quantification of immunoblots underlines the potential of the anti-tumorigenic natural agent Calebin A in CRC cells by specifically targeting NF-κB. Taken together, these results indicate that the anti-tumorigenic effects of Calebin A are mediated, similar to IKK inhibitor BMS-345541, by up-stream suppression of the NF-κB (IKK activation) pathway.

### 2.7. Calebin A-Suppressed Binding of p65-NF-κB to DNA Is Abrogated by Reducing Agent DTT

**Figure 7:**
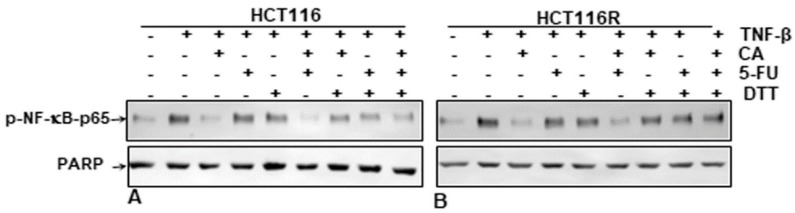
Effect of dithiothreitol (DTT) on the suppressive impact of Calebin A and/or 5-FU in TNF-β-promoted p65-NF-κB interaction to DNA. Extracted nuclei of untreated and Table 116. (**A**) and HCT116R (**B**) cells were co-treated with Calebin A and/or 5-FU in the absence or presence of DTT and then immunoblotted for p65-NF-κB activation. The results are demonstrated from at least three independent assays and the housekeeping protein PARP served as an internal loading control. Densitometric evaluation was performed for p65-NF-κB. * *p* < 0.05, ** *p* < 0.01.

It has been reported previously that several specific blockers of the p65-NF-κB transcription factor compete for the interaction of p65 to DNA because the cysteine residues in the p65 subunit are responsible for its binding with DNA [9,31,32,33,34,35,36]. We investigated if a reduction in cysteine residues by DTT (dithiothreitol) in p65-NF-κB could influence the interaction of p65-NF-κB to the DNA binding in the presence or absence of Calebin A. HCT116 and HCT116R cells were treated as described in the Materials and Methods section. We showed that the suppression of Calebin A on p65-NF-κB interaction to DNA was blocked by DTT in both CRC cell lines (HCT116, HCT116R) (Figure 7A,B), suggesting that Calebin A modulates the interaction of p65-NF-κB to DNA. Taken together, these results indicate the modulation of this important interaction might be one of the most important molecular mechanisms of Calebin A, as it suppresses p65-NF-κB activation.

## 3. Discussion

Colorectal cancer is one of the most frequent causes of cancer-related deaths in the world. Application of chemotherapeutic drugs, like 5-Fluorouracil (5-FU), commonly used in the treatment of colorectal cancer, has become restricted due to negative side effects, drug resistance in almost 50% of cases, cell toxicity and also many patients are non-responders, as they frequently develop metastasis [37,38]. To prevent treatment failure, co-treatment with non-toxic dietary natural anti-cancer agents that possess the capacity to chemosensitize and treat these resistant tumors have great potential [39]. Calebin A, a naturally occurring compound (an ingredient of natural turmeric) has been suggested to have anti-cancer capacity in various solid cancer cells [18,28,29,30].

In this investigation, we examined the therapeutic advantage and the mechanism of Calebin A in the modulation of TNF-β-promoted inflammatory, pro-survival effects in colon cancer resistant and non-resistant cells to 5-FU and in promoting chemosensitization to 5-FU-induced anti-tumorigenic effects.

Chronic inflammation in tissues has been recognized as a major triggering mechanism for the development of cancer [8,9] and previous studies have shown that chronic low-grade inflammation in cancer modulates the interaction between tumor cells and the surrounding tumor environment, thereby promoting malignity of cancer [40,41]. Indeed, environmental stress stimuli account for 95% of cancers as they mediate and trigger chronic inflammation in patients [41]. Intriguingly, several reports indicate that among stimulated pro-inflammatory cytokines, members of the Tumor Necrosis Factor (TNF) family are major players that participate in cancer progression [42,43]. Chronic intestinal inflammation is described to regulate expression and interaction of members of the TNF family such as TNF-α, TNF-β and LTβR acting as major mediators for CRC-related inflammation [44,45]. Moreover, our group has previously shown that TNF-β induces cancer progression and thus stimulates colorectal cancer cell malignity with the same potency as TNF-α [16,18,46]. Further, TNF-β, produced by tumor-associated lymphocytes in the tumor environment, has been shown to promote angiogenesis by signaling through the canonical NF-κB pathway [47], and in Hodgkin lymphoma, autocrine signaling of TNF-β has been described to drive disease progression by sustaining NF-κB activation [46].

In this study, we have demonstrated that Calebin A can induce cell death by apoptosis and inhibit proliferation, colony formation and invasion in both CRC cell lines in a dose- and time-dependent manner. Calebin A, in combination with 5-FU treatment, led to an increase of cell death via apoptosis (apoptotic bodies) and promoted caspase-3 activation in HCT116 and HCT116R cells. The findings indicate strongly that Calebin A pre-treatment can chemosensitize the 5-FU resistant cells to 5-FU, and potentiate apoptosis induced by 5-FU via the cleavage of caspase-3 cascade with mitochondrial changes and fragmentation of nuclei, as shown by transmission electron microscopy. The results further showed that Calebin A could significantly increase all above-mentioned effects in both CRC cells to 5-FU at least in part by suppression of master pro-inflammatory transcription factor, NF-κB and pro-inflammatory agents that are linked with tumor growth by the activation of genes coding for NF-κB-mediated anti-apoptotic and pro-proliferation molecules in the tumor environment. Indeed, it is well established that NF-κB activation and pathway have a key role in mediating cancer drug resistance, the survival, proliferation, migration and angiogenesis of several tumor cells [48,49,50,51], and targeting of NF-κB pathway may play a central role in reversing resistance to chemotherapeutics and enhancing chemosensitization of cancer cells [52,53,54].

Here, we demonstrate, for the first time, results describing that Calebin A exposure in combination with 5-FU has a stronger anti-cancer impact than either agent by itself in CRC cells. Indeed, these results indicate that the significance of Calebin A as a natural compound for the promotion of apoptosis could be used as a supplement with the drug 5-FU, which is routinely employed in the management of colorectal cancer, but is toxic and ineffective in a large majority of patients, as they frequently develop metastasis [37,38].

It is known that cancer cell progression, resistance, recurrence and invasion are driven by a subpopulation of cells in the cancer cell population cancer stem cells (CSC) and they are responsible for the initiation of cancer progression [55,56,57]. Interestingly, previous works have shown that the chemoresistant CRC cells expressed increased several biomarkers of CSC and of epithelial-to-mesenchymal-transition (EMT), a higher capacity to become more tumor malignity (motility, proliferation, drug resistance) and developed colonospheres. It has been demonstrated that other natural compounds like curcumin, resveratrol and/or 5-FU significantly decreased CSC- and EMT-biomarkers expression in CRC cells [16,58,59]. Importantly, it is to be expected that Calebin A as a natural anti-tumor agent has a crucial target on the cancer stem cells in the tumor cell populations.

We found that Calebin A inhibited TNF-β-induced phosphorylation and translocation of p65 from the cytoplasm to the cell nucleus and suppressed NF-κB-regulated tumor-promoting biomarkers that are involved in invasion (MMP-9), metastasis (CXCR4, β1-integrin), proliferation (Ki-67) and upregulated apoptosis (caspase-3). A plethora of studies have shown that activation of the NF-κB pathway plays a crucial role in the development of chronic diseases by stimulating a low-grade chronic inflammatory environment [46,47,60]. Indeed, phosphorylation of NF-κB is the primary step for the induction of these target genes [61]. We have previously demonstrated that TNF-β/NF-κB-induced CRC proliferation, chemoresistance and tumor stem cell promotion was suppressed by natural substances such as resveratrol [16,17]. Furthermore, in a previous study on CRC, we showed the potential of Calebin A on suppressing-TNF-β-induced inflammatory signaling, blocking proliferation, migration and invasion of CRC cells [18], emphasizing the promising of natural substances on targeting TNF-β/NF-κB signaling axis.

Additionally, the TNF-β and/or 5-FU-promoted NF-κB-dependent gene end-products have been shown to associate with tumor cell survival and the development of drug resistance in several tumor cells [62], highlighting the existence of other intracellular pathways, how Calebin A promotes 5-FU-induced apoptosis and, thereby, contributes to the therapeutic effect. Further, we demonstrated that this inhibition was also mediated through suppression of upstream kinase IKK, similar to BMS-345541 (a specific inhibitor of IKK) and, thereby, blocked NF-κB activation in both CRC cells. Moreover, IKK overexpression for the activation of NF-κB is an important mechanism, which is also promoted by proinflammatory cytokines and inhibition of its expression by Calebin A supports the earlier data on its mechanism of action through the NF-κB axis [28,63,64]. Our findings are in accordance with other studies, showing that modification of NF-κB activation at multiple pathway steps, may have the prominent potential for therapeutic applications [65]. Interestingly, it has been previously reported that drugs targeting kinases, which mediate the primary phosphorylation step of NF-κB, may act as promising new agents for adjuvant cancer therapy [62,65].

We could show also that Calebin A suppressed the NF-κB interaction to the DNA to modulate gene transcription. Indeed, a large body of literature previously reported that many inhibitors of the p65-NF-κB transcription factor compete for the binding of p65-NF-κB to DNA and the cysteine residues in the p65-NF-κB subunit are responsible for its direct interaction with DNA [9,18,30,31,32,33,34,35]. Furthermore, related pathways have been reported for plumbagin [9], N-tosyl-L-phenylalanine [33], Bharangin [66], Calebin A [28] and Piperlongumine [34].

**Figure 8:**
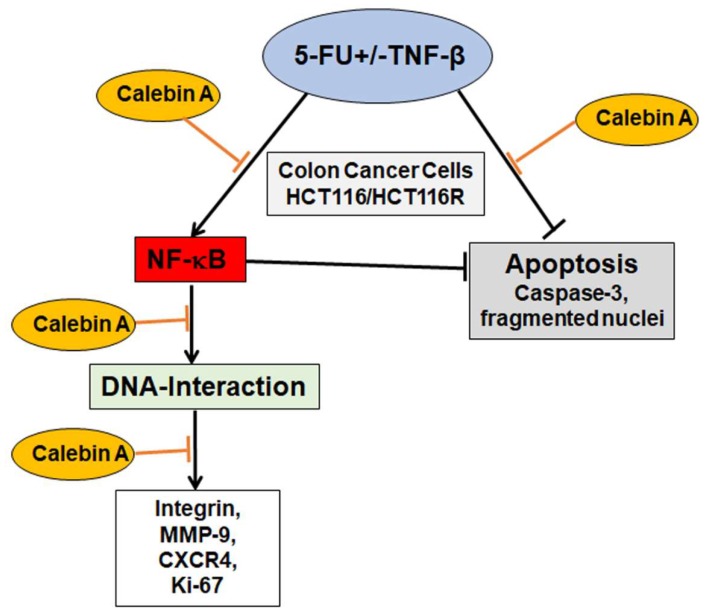
Working model for the modulatory and chemosensitizing effects of Calebin A on the malignity of 5-FU resistant and non-resistant cells by targeting p65-NF-κB activation in TNF-β-promoted viability of CRC cells.

One of the main impediments in cancer treatment regimens is the development of multi-drug resistance leading to decreased apoptosis, enhanced proliferation and metastasis. Indeed, tumor cells are rapidly developing resistance to the majority of chemotherapeutic drugs resulting in tumor recurrence [67,68,69]. Interestingly, it has been shown that an abundance of natural substances may be used as adjuvants in cancer treatment regimens to reverse chemoresistance to chemotherapeutic drugs [70,71]. Several reports from ours and other laboratories have reported that tumor cells can acquire drug resistance by different mechanisms including mutation or overexpression of the drug target, inactivation of the drug or removal of the drug from the cell [16,72,73]. We and others have previously shown for curcumin and for resveratrol potent chemosensitization effects to 5-FU by targeting multiple intracellular signaling pathways (NF-κB, Src, EGF-R, IGF-1R and Akt), thereby suppressing proliferation, colonosphere formation and invasion [16,74,75,76,77]. Calebin A suppressed and synergized with 5-FU in TNF-β-promoted CRC cell proliferation by downregulation of CXCR4, β1-integrin and Ki-67, which are responsible for proliferation and tumor invasion, highlighting the tremendous multitargeting potential role of Calebin A in the prevention of cancer cell proliferation, invasion, metastasis and apoptosis (Figure 8).

Based on our investigations, we can conclude that these findings provide molecular evidence that Calebin A renders 5-FU chemoresistant cells sensitive to 5-FU. Further, Calebin A, in combination with 5-FU, represents a potential conventional treatment option for resistant colon cancer cells by suppression of NF-κB, which is promoted by chemotherapeutic drugs and proinflammatory agents.

## 4. Materials and Methods

### 4.1. Antibodies and Chemicals

Monoclonal antibodies to p65, as well as phospho-specific p65-NF-κB, MMP-9, CXCR4, cleaved-Caspase-3, β1-Integrin and PARP, were obtained from R&D Systems (Heidelberg, Germany). Antibodies to β-Actin were from Sigma-Aldrich (Taufkirchen, Germany). Secondary rhodamine-coupled antibodies for immunofluorescence and anti-Ki67 were from Dianova (Hamburg, Germany) and alkaline phosphatase-linked antibodies for Western blotting from EMD Millipore (Schwalbach, Germany). MTT reagent (3-(4,5-dimethylthiazol-2-yl)-2,5-diphenyltetrazolium bromide), DAPI, 5-Fluorouracil (5-FU), alginate, BMS-345541 and dithiothreitol (DTT) were from Sigma-Aldrich (Taufkirchen, Germany). Stock solutions of BMS-345541 (1000 µM) and of DTT (1000 mM) were prepared in PBS and further diluted in normal cell culture growth medium to obtain final concentrations. Then, 5-FU was diluted as a 1000 µM stock solution in ethanol and further diluted in the cell culture medium. Final concentrations of ethanol did not exceed 0.1% during treatment. TNF-β was purchased from eBiosciences (Frankfurt, Germany). Further, TNF-β was given as a kind gift by Genetech (South San Francisco, CA, USA). Calebin A (CA), with a purity of 99.65%, was a generous gift from Sabinsa Corporation (East Windsor, NJ, USA). Epon for electron microscopy was purchased from Plano (Marburg, Germany).

### 4.2. Cells and Cell Culture Conditions

The colorectal cancer (CRC) cell line HCT116 was obtained from the European Collection of Cell Cultures (Salisbury, UK). To investigate the effect of Calebin A also on chemoresistant CRC cells, we additionally evaluated 5-Fluorouracil (5-FU) resistant derivatives of the HCT116 cell line, referred to as HCT116R, which was a generous gift from our cooperation partner Prof. Dr. Ajay Goel from the Department of Molecular Diagnostics and Experimental Therapeutics, Beckman Research Institute (Duarte, CA, USA) [16,74,78]. The chemoresistant cell line was created in his laboratory by successive treatment of the cells with an increasing concentration of 5-FU over a one-year period.

### 4.3. Study Design

CRC cells were cultured in a humidified incubator (37 °C, 5% CO_2_) with cell culture growth medium containing 10% FCS as described before [77]. To elucidate the mechanism of chemosensitization potential of Calebin A on CRC cells towards 5-FU in the TNF-β-stimulated inflammatory environment, experiments were performed in monolayer or three-dimensional alginate tumor environment culture in vitro. Hereby, the three-dimensional alginate culture provides a close to in vivo tumor environment, which is very appropriate to study early events in cancerogenesis [73,77,78]. All experiments were performed in a cell culture medium containing only 3% FCS, and before starting the actual experiments, cells were incubated for 1 h with serum reduced medium.

### 4.4. Vitality Assay

Cell growth and vitality were examined by the MTT method as described in detail before with a revelation scanner (Biorad, Munich, Germany) at a 550 nm optical density [17]. For monolayer investigation, CRC cells were left untreated and/or treated with TNF-β (10 ng/mL), Calebin A (2, 5 µM), 5-FU (1, 2 nM) alone or in combination with 5-FU (2 nM) and TNF-β (10 ng/mL) and Calebin A (5 µM), or with Calebin A (5 µM) and TNF-β (10 ng/mL) or with Calebin A (5 µM) and 5-FU (2 nM) and TNF-β (10 ng/mL) for 3 days.

### 4.5. Migration and Colony Formation Assay

Chemosensitization effect of Calebin A on 5-FU and TNF-β stimulated proliferation, invasion and colony formation of HCT116 and HCT116R cells were investigated in three-dimensional alginate bead cultures as described in detail before [18]. Cells were left untreated, and/or treated with TNF-β (10 ng/mL), Calebin A (5 µM), 5-FU (2 nM) alone, or with 5-FU (2 nM) and either TNF-β (10 ng/mL), or Calebin A (5 µM), or with Calebin A (5 µM) and TNF-β (10 ng/mL) or with Calebin A (5 µM) and 5-FU (2 nM) and TNF-β (10 ng/mL) for 10 days. On day 10, images of alginate beads were captured, and emigrated colonies on the bottom of the Petri dishes were stained with toluidine blue, images captured and colonies finally quantified under a light microscope (Zeiss, Germany).

### 4.6. Ultrastructural Investigations

To investigate apoptosis on an ultrastructural level, CRC cells in the monolayer culture were left untreated, or were treated as described above for 3 days. As described in detail before [77], samples were fixed with a karnowsky fixative for 1 h, post-fixated with OsO_4_ for 2 h, dehydrated in an ascending series of alcohols and finally embedded in Epon. Ultrathin sections were prepared and evaluated with a transmission electron microscope (Jeol 1200 EXII, Akishima Tokyo, Japan (Institute of Pathology, Technical University of Munich, Germany). Quantification of apoptosis was performed by counting 800–1000 cells from 30 microscopic fields.

### 4.7. Immunofluorescence Labeling

CRC cells in the monolayer culture were left untreated and/or treated as described in earlier sections for 4 h. For immunofluorescence investigations of p65-NF-κB, cultures were incubated for 2 h with primary antibody (1:100 in PBS), washed three times with PBS and incubated for 2 h with rhodamine-coupled secondary antibody (1:100 in PBS) at ambient temperature. To detect nuclei, cultures were additionally incubated with DAPI for 15 min. Staining was investigated with a Leica DM2000 microscope (Wetzlar, Germany) and images digitally captured and stored as previously described [78]. Quantification of positively p65-NF-κB-labeled nuclei and apoptotic cells was performed by counting 500–800 cells from 20 different microscopic fields.

### 4.8. Immunoblotting

Immunoblotting of whole-cell lysates was performed as described in detail [77,78]. CRC cells in the monolayer culture were left untreated, and/or treated with TNF-β (10 ng/mL), Calebin A (2, 5 µM) and 5-FU (1, 2 nM) alone, or in combination with 5-FU (2 nM) and either TNF-β (10 ng/mL) and Calebin A (2, 5 µM), or with Calebin A (2, 5 µM) and 5-FU (2 nM) and TNF-β (10 ng/mL) for 3 days. In an additional experiment, CRC cells (HCT116, HCT116R) in the monolayer culture were left untreated, and/or treated with TNF-β (10 ng/mL), Calebin A (2 µM), specific IKK inhibitor BMS-345541 (5 µM) and 5-FU (2 nM) alone, or in various combinations for 4 h. Briefly, whole-cell lysates were separated by SDS-PAGE electrophoresis under reducing conditions and samples were blotted onto a nitrocellulose membrane using a transblot apparatus (Biorad, Munich, Germany). All experiments were performed at least three times, anti-β-actin served as an internal control and to normalize the sample amounts and densitometric quantification was performed with the Quantity One program (Biorad, Munich, Germany). For the DNA-binding assay, serum-starved HCT116 and HCT116R cells in the monolayer were left untreated and/or pre-treated for 4 h with TNF-β (10 ng/mL). The nuclei were isolated and left untreated or incubated additionally 1 h with Calebin A (5 µM), 5-FU (2 nM) or DTT (5 mM) alone, or in combinations of either Calebin A and/or 5FU and/or DTT. Finally, nuclear extracts were prepared for immunoblotting and analyzed for NF-κB activation.

### 4.9. Statistical Evaluation

All assays were performed three times as an individual assay with three different replicates. For statistical analysis, a Wilcoxon–Mann–Whitney test was applied. Results were shown as mean values ± SD or SEM and were compared by a one-way, two-way or three-way ANOVA using SPSS Statistics, if the normality test passed (Kolmogorov–Smirnov test), followed by Tukey’s post hoc test were used. The pairwise equation with control and other groups showed highly significant. *p* < 0.05 was considered to establish statistically significant differences.
